# Paraspinal muscle changes after single-level posterior lumbar fusion: volumetric analyses and literature review

**DOI:** 10.1186/s12891-020-3104-0

**Published:** 2020-02-05

**Authors:** Sung-Min Cho, Se-Hoon Kim, Sung-Kon Ha, Sang-Dae Kim, Dong-Jun Lim, Jaehyung Cha, Bum-Joon Kim

**Affiliations:** 1 Department of Neurosurgery, Korea University Ansan Hospital, 123 Jeokgeum-ro, Danwon-gu, Ansan, Gyeonggi-do 15355 South Korea; 2 Medical Science Research Center, Ansan Hospital, Korea University College of Medicine, Ansan, South Korea

**Keywords:** Lumbar spinal fusion, Paraspinal muscle atrophy, Denervation, Multifidus, Back muscles

## Abstract

**Background:**

Posterior lumbar fusion is a widely accepted surgical technique; however, it has been related to the possibility of paraspinal muscle atrophy after surgery. We investigated 1-year postoperative changes in paraspinal muscle volume using a simple formula applicable to magnetic resonance imaging (MRI) or computed tomography (CT) images.

**Methods:**

Patients with degenerative lumbar spinal stenosis who underwent posterior interbody fusion (PLIF) at the L4/5 level in the period from May 2010 to June 2017 were enrolled in this study. Radiologic parameters were measured using MRI or CT images which were taken before surgery and at 1 year after surgery. The volume of the paraspinal muscles was calculated using a simple formula which was derived from the formula for calculating the volume of truncated elliptic cones.

**Results:**

A total of 40 patients were included; 24 were analyzed using MRI and 16 were analyzed using CT. The mean age of the patients was 59.6 ± 12.1 years and 32 (80.5%) were female. When comparing the preoperative and 1-year-postoperative images, multifidus muscle (MF) reduction was consistently observed in the MRI and CT groups, right and left (*p* = 0.003, *p* < 0.001, *p* = 0.005 and *p* < 0.001, respectively). In the erector spinae (ES) group, decrease in muscle volume was observed in the right-sided muscles of the CT group (*p* < 0.001), but no significant change was observed in the MRI group. The psoas muscle showed no significant change after 1 year. Conversely, regression analysis showed a negative correlation between MF muscle volume loss and age in the MRI group (right and left, *p* = 0.002 and *p* = 0.015, respectively), that is, the younger the age, the greater loss of muscle mass.

**Conclusion:**

After the posterior lumbar fusion, the volume of the MF muscles was markedly decreased, and the degree of decrease was apparent in the MRI. The volume of the ES muscles, which are located relatively laterally, also tended to decrease at 1 year after surgery.

## Background

South Korea has become an aged society and the incidence of spinal stenosis is also increasing [1]. Posterior lumbar fusion surgery is a widely accepted surgical technique in the treatment of lumbar spinal stenosis. However, this procedure is known to have several disadvantages, one of which is the postoperative atrophy of the paraspinal muscles [2–6]. Minimally invasive spinal surgery techniques have been developed to reduce muscle injuries [7, 8]; however, these injuries remain common in clinical practice.

Most previous studies explored the effects of posterior lumbar fusion surgery on the paraspinal muscles by quantitative analysis of magnetic resonance imaging (MRI) or computed tomography (CT) images using specific software [4, 5, 9–12]. However, the use of this software requires proficiency and it is therefore cumbersome to use it in clinical practice. There are also many studies wherein two-dimensional analysis was performed through measurement of the cross-sectional area [2, 5, 8, 11, 12]. However, the accuracy of the cross-section seems to be limited considering that the cross-section may not be uniform during MRI.

We have designed a simple formula that allows volumetric measurement using MRI and CT images without any special software. Herein, we report the postoperative muscle changes measured using this formula.

## Materials and methods

### Patients and method

Patients diagnosed with degenerative lumbar spinal stenosis who underwent posterior lumbar interbody fusion (PLIF) surgery at the L4/5 level in the period from May 2010 to June 2017 were included in this study. The exclusion criteria were as follows: multi-level surgery, trauma, history of previous back surgery, tumor, infection, and no adequate preoperative and 1-year-post-surgery MRI or CT images.

### Operation technique

Surgery was performed in the prone position under general anesthesia. A midline skin incision was made from the L4 to L5 spinous processes; the paraspinal muscles were stripped bilaterally using a monopolar electrical cautery. After a fusion retractor was applied, subtotal laminectomy with medial bilateral facetectomies were performed. Subsequently, two polyetheretherketone (PEEK) cages filled with local autologous bone chips and demineralized bone matrix (DBM) were inserted into the L4/5 intervertebral space. Four pedicle screws were inserted and assembled with two rods. All patients received the same postoperative treatment, with ambulation starting on the second postoperative day, application of a brace for 6 weeks, and no special rehabilitation exercises.

### Acquisition of data

**Fig. 1:**
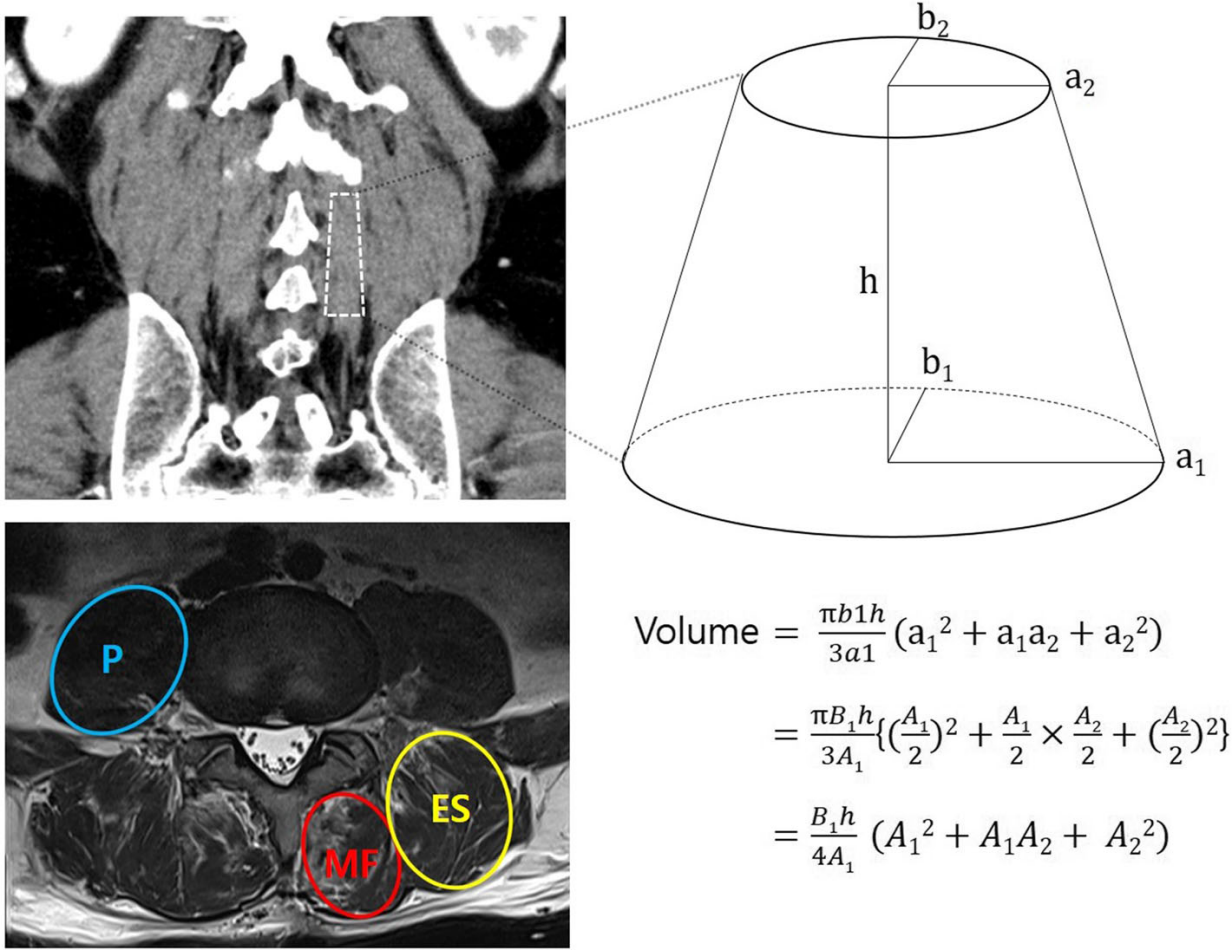
Measurements of paraspinal muscles. MF, multifidus; ES, erector spinae; P, psoas. a = semi-major axis (cm). b = semi-minor axis which is perpendicular to the semi-major axis (cm). A = maximum muscle diameter (cm). B = maximum diameter perpendicular to A on the same slice (cm). h = vertical height (cm)

The clinical and radiological data were collected in accordance with the regulations of the institutional review board at our hospital. The radiologic parameters were measured in MRI or CT images obtained preoperatively and at 1 year postoperatively. The formula for the volume measurement of the paraspinal muscles was used to calculate the volume through a simple measurement of the image, similar to the ABC / 2 formula used to measure the intracerebral hemorrhage volume. As shown in Fig. 1, this formula was derived from the formula used to calculate the volume of truncated elliptic cones [13]. The area of the upper surface of the truncated elliptic cone for muscle volume calculation was measured as the cross-sectional area on the axial MRI or CT image taken at the L3 lower endplate level (Fig. 1.). Similarly, the lower surface was measured at the upper endplate level of S1. The height of this three-dimensional figure was defined as the distance between the L3 lower endplate and the upper endplate of S1 in the mid-sagittal image. If a preoperative MRI was performed at baseline then an MRI was used in the follow up. Conversely, if a preoperative CT was performed at baseline then a CT was used in the follow up. The parameters measured in preoperative imaging were compared with those measured in the images obtained 1 year after the surgery.

### Statistical analysis

For the comparison of the MRI group and the CT group, chi-square test was used for categorical variables and Student t-test or Mann-Whitney U test for continuous variables. Comparisons of the muscle volumes between preoperative and 1 year post-surgery images were made using paired t-test with Bonferroni post-hoc correction. However, Wilcoxon signed ranks test was used for the right psoas muscle because it did not follow a normal distribution. To assess the correlation of changes in the muscle volume with the patients’ age as well as with body mass index (BMI), regression analyses were performed. Statistical calculations were performed using SPSS software, version 20.0 (IBM Corp., Armonk, NY, USA).

## Results

Of the 112 patients who underwent posterior lumbar interbody fusion (PLIF) surgery at the L4/5 level, 40 patients were included in the study (72 patients were excluded after applying the exclusion criteria). Of these 40 patients, 24 patients had undergone MRI and 16 patients had undergone CT (Table 1), both before surgery and 1 year after surgery. The mean age of the patients was 59.6 years and 32 (80.0%) were female. There was no significant difference in gender distribution, mean age, mean BMI and the measured preoperative volume of paraspinal muscles between the MRI group and the CT group (Table 1). On the other hand, the muscle volume measured 1 year after surgery in the right-sided multifidus muscle (MF) was smaller in the MRI group (*p* = 0.034) and that measured in the bilateral erector spinae muscles (ES) was smaller in the CT group (right and left, *p* = 0.002 and *p* = 0.020, respectively).

**Table 1:** Comparison of overall parameters between MRI and CT groups

		MRI (*n* = 24)	CT (*n* = 16)	*p*-value
Female, n (%)		13 (54.2%)	9 (56.3%)	0.897
Age, mean ± SD		61.79 ± 12.13	58.19 ± 12.04	0.362
BMI (kg/m^2^)		24.53 ± 3.61	25.46 ± 3.71	0.433
Preoperative, mean (cm^3^ ± SD)				
MF	Right	33.08 ± 3.89	34.25 ± 3.65	0.835
	Left	33.83 ± 3.45	34.67 ± 3.53	0.870
ES	Right	90.83 ± 6.79	71.47 ± 9.05	0.090
	Left	85.24 ± 6.45	66.15 ± 9.70	0.097
Psoas	Right	59.96 ± NA	68.25 ± NA	0.420^b^
	Left	63.59 ± 5.24	63.79 ± 7.08	0.982
Height of vertebrae		5.73 ± 0.48	5.58 ± 0.39	0.306
1 year after PLIF, mean (cm^3^ ± SD)				
MF	Right	19.66 ± 1.69	27.63 ± 3.65	0.034^a^
	Left	20.11 ± 2.08	26.71 ± 3.28	0.082
ES	Right	82.90 ± 6.96	49.54 ± 6.88	0.002^a^
	Left	78.76 ± 6.19	53.75 ± 8.60	0.020^a^
Psoas	Right	59.82 ± NA	72.77 ± NA	0.202^b^
	Left	64.41 ± 5.93	67.60 ± 7.44	0.738
Height of vertebrae		5.81 ± 0.38	5.63 ± 0.37	0.148

*MRI* magnetic resonance imaging, *CT* computed tomography, *BMI* body mass index, *SD* standard deviation, *MF* multifidus, *ES* erector spinae, *NA* not applicable

^a^ Statistically significant difference was defined as *p* < 0.05

^b^ Mann–Whitney U test was used because the data was not normally distributed

**Fig. 2:**
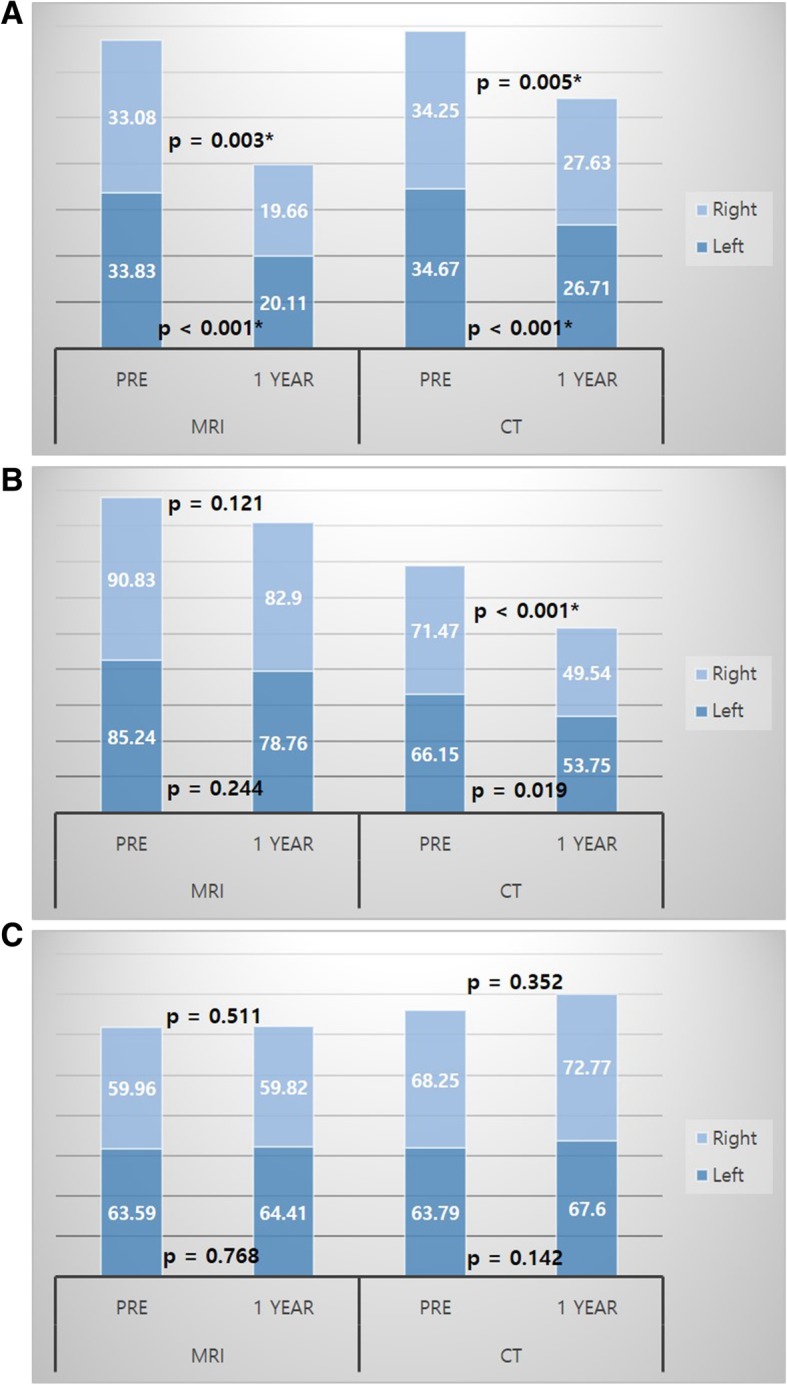
Histograms of the sum of right and left paraspinal muscles on MRI and CT. **a** Volume of the multifidus muscles. **b** Volume of the erector spinae muscles. **c** Volume of the psoas muscles

As shown in Table 2, although there was no statistically significant difference (*p* > 0.05), the height of the vertebrae (the distance between the L3 lower endplate and the S1 upper endplate) increased by 0.5–0.8 mm in both the MRI and CT groups. However, when the paraspinal muscle volume was compared between the preoperative and postoperative images, there was a postoperative reduction of the MF, and this was consistently observed in the right and left side of both the MRI and the CT groups (Fig. 2a; *p* = 0.003, *p* < 0.001, *p* = 0.005 and *p* < 0.001, respectively). On the other hand, no significant change in the ES volume was observed in the MRI group; however, a decrease in the muscle volume was observed in the right-sided ES in the CT group (Table 2 and Fig. 2b, *p* < 0.001). There was no significant postoperative change in the psoas muscle volume in both the MRI and CT groups (Table 2 and Fig. 2c). Comparison of the change in volume of each analyzed paraspinal muscle presented in Fig. 2 (a, b, c) revealed that the volumes of the MF and ES tended to decrease overall, while the volume of the psoas muscles tended to be unchanged, or even increased.

**Table 2:** Comparative analysis of pre- versus post-operative paraspinal muscle volume on MRI and CT

	MRI (*n* = 24)		*p*-value	CT (*n* = 16)		*p*-value
	pre	post		pre	post	
MF (cm^3^ ± SD)						
Right	33.08 ± 3.89	19.66 ± 1.69	0.003^a^	34.25 ± 3.65	27.63 ± 3.65	0.005^a^
Left	33.83 ± 3.45	20.11 ± 2.08	< 0.001^a^	34.67 ± 3.53	26.71 ± 3.28	< 0.001^a^
ES (cm^3^ ± SD)						
Right	90.83 ± 6.79	82.90 ± 6.96	0.121	71.47 ± 9.05	49.54 ± 6.88	< 0.001^a^
Left	85.24 ± 6.45	78.76 ± 6.19	0.244	66.15 ± 9.70	53.75 ± 8.60	0.019
Psoas (cm^3^ ± SD)						
Right	59.96 ± NA	59.82 ± NA	0.511^b^	68.25 ± NA	72.77 ± NA	0.352^b^
Left	63.59 ± 5.24	64.41 ± 5.93	0.768	63.79 ± 7.08	67.60 ± 7.44	0.142
Height of vertebrae	5.73 ± 0.48	5.81 ± 0.38	0.177	5.58 ± 0.39	5.63 ± 0.37	0.181

*MRI* magnetic resonance imaging, *CT* computed tomography, *SD*, standard deviation, *MF* multifidus, *ES* erector spinae, *NA* not applicable

^a^ Statistically significant difference was defined as *p* < 0.00714 (0.05/7) by Bonferroni correction

^b^ Wilcoxon signed ranks test was used because the data was not normally distributed

In the regression analysis, the postoperative volume loss of both the right-sided and left-sided MF muscles showed a negative correlation with the age of the patients in the MRI groups (*p* = 0.002 and *p* = 0.015, respectively). The volume loss of the right ES in the CT group was negatively correlated with the age of the patients (*p* = 0.016) (Table 3); therefore, our data suggests that the younger patients experienced the greatest loss of muscle mass. In contrast, the BMI was not correlated with the changes in muscle volume in both the MRI and CT groups (Table 4). Although we have not included this data, we also conducted regression analysis to determine the correlation between the gender and the changes in muscle volume; no significant correlation was observed.

**Table 3:** Regression analysis of the relationship between the postoperative paraspinal muscle volume loss and age

Age vs	MRI (*n* = 24)		*p*-value	CT (*n* = 16)		*p*-value
	β coefficient	Adjusted R^2^		β coefficient	Adjusted R^2^	
Changes of MF (cm^3^)						
Right	−0.592	0.321	0.002^a^	−0.076	− 0.065	0.780
Left	− 0.489	0.204	0.015^a^	0.114	−0.057	0.674
Changes of ES (cm^3^)						
Right	0.203	−0.002	0.342	−0.590	0.301	0.016^a^
Left	0.267	0.029	0.207	−0.281	0.013	0.292
Changes of Psoas (cm^3^)						
Right	0.184	−0.010	0.388	0.019	−0.071	0.945
Left	0.155	−0.020	0.469	0.189	−0.033	0.482

*MRI* magnetic resonance imaging, *CT* computed tomography, *SD* standard deviation, *MF* multifidus, *ES* erector spinae

^a^Statistically significant difference was defined as *p* < 0.05

**Table 4:** Regression analysis of the relationship between the postoperative paraspinal muscle volume loss and BMI

BMI vs	MRI (*n* = 24)		*p*-value	CT (*n* = 16)		*p*-value
	β coefficient	Adjusted R^2^		β coefficient	Adjusted R^2^	
Changes of MF (cm^3^)						
Right	−0.371	0.098	0.075	−0.004	−0.071	0.987
Left	−0.214	0.002	0.315	0.293	0.021	0.271
Changes of ES (cm^3^)						
Right	0.184	−0.010	0.391	−0.128	− 0.054	0.636
Left	0.009	−0.045	0.965	−0.329	0.044	0.214
Changes of Psoas (cm^3^)						
Right	−0.057	−0.042	0.793	−0.083	− 0.064	0.759
Left	0.107	−0.034	0.619	0.178	−0.038	0.511

*BMI* body mass index, *MRI* magnetic resonance imaging, *CT* computed tomography, *SD* standard deviation, *MF* multifidus, *ES* erector spinae

^*^ Statistically significant difference was defined as *p* < 0.05

## Discussion

It has been reported by many researchers that back muscle atrophy is clinically associated with lower back pain (LBP) and radiculopathy [11, 12, 14–19]. Mengiardi et al. found that there was a higher proportion of fat content in the MF muscles in patients with chronic LBP [17], and Hyun et al. suggested the possibility of denervation-related atrophy in lumbosacral radiculopathy [11]. Rantanen et al. suggested that when disc herniation compresses the spinal nerve, it also compresses the dorsal rami innervating the MF and ES, thereby causing muscle damage [16], and they asserted through the MF biopsy study that these muscle changes can be reversed by appropriate surgical treatment [16]. We investigated whether the paraspinal muscle atrophy occurs after single level lumbar fusion and reviewed our findings in light of previous literature.

There are many reports of postoperative paraspinal muscle atrophy associated with posterior lumbar surgery. Sihvonen et al. pointed out that the cause of postoperative atrophy was iatrogenic denervation of the paraspinal muscles during lumbar surgery [20]. However, Tsutsumimoto et al. claimed that mini-open PLIF is preferable because injury of the medial MF of the paraspinal muscle is caused by direct injury from dissection or retraction [4]. Kim et al. suggested that percutaneous screw fixation causes less muscle atrophy and is more beneficial than open pedicle screw fixation for trunk muscle performance after surgery [2]. Tabaraee et al. suggested that ipsilateral MF changes were significantly higher than contralateral in the quantitative study of multifidus muscle changes after minimally invasive (MIS) lumbar discectomy [8]. These findings suggest that less invasive spinal surgery, such as unilateral approach bilateral decompression, may have practical benefits in muscle preservation. In patients with lumbar spinal stenosis who require spinal instrumentation owing to distinct dynamic components or overt instability, utilizing the anterior approach can reduce MF injury. However, considering that the anterior approach is a technically demanding and potentially risky procedure for vascular injury, PLIF remains a useful surgical method in cases where major vessels are found to interfere with the anterior trajectory on imaging studies. Conversely, efforts to reduce muscle atrophy during PLIF surgery, such as reducing paraspinal muscle detachment or minimizing the use of an electric coagulator, are required.

In the current study, atrophy of the MF was prominent; this was consistent with the findings of previous studies. Because the MF is an important stabilizer of the lumbar spine, MF atrophy is considered to be related to LBP [19]. In our study, the association between LBP and severe MF atrophy during the one-year follow-up was unclear. Considering the stabilizing effect of the PLIF surgery, it is difficult to observe the correlation between muscle atrophy and LBP in the short term follow-up. However, given that previous studies have stated that the reduction of paraspinal muscle volume is associated with pain [11, 12, 14–19], the apparent post-operative MF atrophy observed in our study may have a negative impact on the long-term clinical results. On the other hand, the tendency of the ES to decrease in volume overall was observed (Fig. 2b and Table 2). Airaksinen et al. noted that both the psoas and extensor muscles reduced in size in CT studies after laminectomy and facetectomy, and that in addition to denervation, disuse or inactivity induced these atrophies [21]. Although larger studies are required to validate these results, we should consider the effects of motion reduction after fusion and 6 weeks of immobilization with bracing, and not just direct injury or denervation, as causes of muscle atrophy.

MRI is an excellent tool for differentiation of the soft tissues. In particular, in atrophic muscle changes, size is reduced and fat deposits are increased [12, 22]. MRI is superior to CT in the visualization of the intervening fat component between the muscle fibers. In the current study, the MF muscle showed a significantly decreased volume on both the MRI and CT images. In comparison of the MRI and CT group, both groups showed similar measurements before surgery (Table 1). At 1 year after surgery, the volume of the MF muscle decreased by 41.6% ~ 49.6% in the MRI group, while the decrease was 19.3% ~ 23.0% in the CT group (Table 2). MRI is more advantageous in the differentiation of fat signal [12, 22], so it is thought that the degree of fat tissue replacement after denervation injury is more accurately reflected by MRI than by CT. However, in South Korea, the cost of an MRI is 3–4 times that of a CT; therefore, there is a cost disadvantage for monitoring the muscle status with MRI, and the instrumentation may interfere with accurate muscle demarcation due to metallic artifacts. CT is beneficial in terms of the cost and time required for testing, however, it requires the patient to be exposed to radiation. Therefore, this study has some limitations due to its retrospective design, because of which we could not perform pre and post-operative CT and MRI examinations on all the PLIF patients. It was difficult to perform MRI follow-up after surgery in lower income patients, and CT could not be repeatedly performed if the patients did not consent to repeated exposure to radiation.

The advantage of our study is that it can be easily applied at any time in clinical practice because we measured the muscle volume using MRI and CT scans, which are commonly used in practice, without the help of any special software or image processing device. This formula is even more meaningful when considering the fact that in MRI, axial imaging is performed only at regions of interest and the angle of the cross-section is not constant. Additional information on the effects of denervation and immobilization is needed through further analysis with pre and post-operative electromyography of the relevant muscle groups, comparison with unilateral approach surgery, and comparison with anterior fusion and posterior percutaneous screw. Undoubtedly, future studies should focus on the surgical techniques required to preserve the paraspinal muscles after single-level primary lumbar fusion.

## Conclusions

We observed that the volume of the MF muscles was reduced after lumbar fusion surgery using a novel and simple formula. After the posterior lumbar fusion, the volume of the MF muscles, which constitute the medial part of the paraspinal muscles of the operative segment, was markedly decreased, and the degree of the decrease was apparent in the MRI images. Moreover, the volume of the ES muscles also decreased. Because ES muscles are located relatively laterally to the MF muscles and did not suffer direct injury, it is considered that the volume changes might be the result of denervation and immobilization.

## Data Availability

The datasets used and/or analysed during the current study are available from the corresponding author on reasonable request.

## Abbreviations

BMI: Body mass index
CT: Computed tomography
DBM: Demineralized bone matrix
ES: Erector spinae
LBP: Lower back pain
MF: Multifidus
MIS: Minimally invasive surgery
MRI: Magnetic resonance imaging;
PEEK: Polyetheretherketone
PLIF: Posterior lumbar interbody fusion
